# Finding the SNARC Instead of Hunting It: A 20^∗^20 Monte Carlo Investigation

**DOI:** 10.3389/fpsyg.2017.01194

**Published:** 2017-07-18

**Authors:** Krzysztof Cipora, Guilherme Wood

**Affiliations:** ^1^Department of Psychology, University of Tübingen Tübingen, Germany; ^2^Institute of Psychology, Karl-Franzens-University of Graz Graz, Austria

**Keywords:** SNARC effect, Monte Carlo simulations, power analysis, ANOVA, Regression Analysis

## Abstract

The Spatial Numerical Association of Response Codes (SNARC) effect describes a stimulus-response association of left with small magnitude and right with large magnitude. Usually, it is estimated by means of regression slopes, where the independent variable only has a limited number of levels. Inspection of the literature reveals that it is not difficult to detect a SNARC effect within a group, but it has been quite unusual to find group differences. Is the SNARC effect as it is usually estimated using regression slopes largely insensitive to group differences, and are there design parameters necessary to increase sensitivity in group comparison analyses? Using numerical simulations, we provide evidence that both sample size and the number of stimulus repetitions, as well as intra-individual variability, contribute in a substantial way to the probability of detecting an existing SNARC effect. Our results show that the adequate choice of either sample size or number of repetitions per experimental cell does not fully compensate for a poor choice of the other parameter. Moreover, repeated failures to find significant group differences in the SNARC effect can be explained by insufficient power. Fortunately, increasing the number of repetitions to about 20 and testing at least 20 participants provides in most cases sufficient sensitivity to reliably detect the SNARC effect as well as group differences. Power plots are provided, which may help to improve both the economy and sensitivity of experimental design in future SNARC experiments, or, more generally when regression slopes are estimated intra-individually.

## Introduction

Several studies show an interaction between number magnitude and side of response. Small magnitude numbers are responded to faster on the left-hand side whereas large magnitude numbers are responded to faster on the right-hand side. To name this phenomenon, the acronym SNARC (Spatial Numerical Association of Response Codes) was coined (Dehaene et al., 1993).

The classical method to estimate the SNARC effect was proposed by Fias et al. (1996) and generalized lately (Pinhas et al., 2012; Tzelgov et al., 2013). The SNARC can be described as the relation between number magnitude and reaction time differences – dRTs estimated separately for each individual. dRTs are calculated by subtracting averaged left-hand RTs from averaged right-hand RTs for each number. Negative dRT values indicate faster right hand responses compared to left hand responses. In the next step, dRTs are regressed on number magnitude using the least squares method. It is done for each participant separately. The regression slope is interpreted then as a measure of the SNARC effect in a given subject. To test whether the SNARC effect is significant in the sample under investigation, individual standardized or non-standardized slopes are compared to 0 by means of a one-sample *t*-test (see generalization of this approach to more complex experimental designs by Pinhas et al., 2012 and Tzelgov et al., 2013).

Such a measure is considered to be convenient because (1) it describes the SNARC association with a single numerical parameter, (2) it allows simple categorization as to whether or not a given participant presents the SNARC, (3) it can be simply interpreted (e.g., non-standardized slope = -5 means that a one-unit increase in magnitude leads to a 5 ms advantage in the right hand response compared to the left hand response). Frequently, reported slopes in studies examining healthy adults vary from -10 ms (Castronovo and Seron, 2007, Exp. 1, control group) to -3 ms (Nuerk et al., 2005).

In several experiments, the SNARC effect has been used to investigate other processes of numerical cognition. For instance, it served as a measure of semantic processing of numbers (e.g., Fias et al., 1996, 2001; Lammertyn et al., 2002). Moreover, the SNARC effect was employed to differentiate between mathematically skilled and non-skilled participants (Dehaene et al., 1993, Exp. 1; Fischer and Rottmann, 2005, Exp. 1; Hoffmann et al., 2014; Cipora et al., 2016; but see: Bonato et al., 2007, Exp. 1 and 4; Bull et al., 2013; Cipora and Nuerk, 2013). Furthermore, gender differences in the SNARC effect were also reported (Bull et al., 2013). Attempts were made to use the SNARC effect as a diagnostic tool for screening mathematical difficulties and impairments in visuospatial processing in school children (Bachot et al., 2005; van Galen and Reitsma, 2008; Schneider et al., 2009) as well as to investigate the development of the number parity concept (Berch et al., 1999). In several studies, the SNARC effect was employed as a measure of numerical processing that differentiates number processing in control participants from subjects with a neurological or sensory impairment, such as (1) patients with neglect (Vuilleumier et al., 2004; Priftis et al., 2006), (2) blindness (Castronovo and Seron, 2007), and (3) deafness (Bull et al., 2005, 2006). Moreover, the SNARC effect was also used as a measure of spatial representation of magnitude in a study investigating differences in spatial processing related to hormonal masculinization (Bull and Benson, 2006) or the differences in spatial mapping of numbers depending on cultural influences, namely the reading direction (Shaki et al., 2009). These empirical examples as well as a quantitative meta-analysis (Wood et al., 2008) and more recent reviews (Fischer and Shaki, 2014; Winter et al., 2015) on spatial-numerical associations point out the strength of such individual and group differences in the SNARC effect. The mechanisms responsible for these differences are still poorly understood and may require a refinement of the measurement strategies employed to design SNARC experiments targeting within and between subject comparisons. More specifically, the determinants of SNARC effect detection in within- and between-subject designs as well as the requirements for designing statistically fair experiments have to be investigated and their relevance for empirical studies quantified.

One large step made by Pinhas et al. (2012) and Tzelgov et al. (2013) was to redefine the estimation of individual slopes in the context of mixed-effects models, which may test simultaneously and appropriately for within- and between-subject effects.

In the present study, we investigate the role of sources of statistical bias paramount to establish cognitive models and reproducible statistical inferences using the classical estimation of the SNARC effect (Fias et al., 1996) or more advanced statistical models (Pinhas et al., 2012; Tzelgov et al., 2013). These sources of statistical biases are the following: (1) number of replications per item in an experimental session (which then leads to larger values of standard errors), (2) sample size, and (3) intra-individual variability. Moreover, the power to detect a significant difference between two groups regarding the SNARC effect depends on the absolute numerical size of the difference between them.

The current study aims to examine the impact of these experimental factors on the power to detect an existing SNARC effect within-group as well as to detect differences in the SNARC effect between-groups. We ran a series of simulations in which the sample size, number of repetitions of each number, absolute size of the SNARC effect and the variability of intra-individual responses were varied systematically.

## Materials and Methods

The effect of the four parameters, (1) slope size (*sl*)/difference between slopes (*sld*), (2) sample size (*n*), (3) number of repetitions per stimulus within-condition (*k*), and (4) intra-individual variability in reaction time (*sdR*; i.e., the average standard deviation for single items) were investigated. We tested how these parameters influence the probability to detect the SNARC effect (Simulation 1) as well as the probability to detect an existing difference in the SNARC effect between two groups (Simulation 2).

### Parameter Definition

Data were simulated with parameters set at levels typical for empirical studies reported in the literature. Sample sizes (*n*) vary from less than 10 (in studies with groups of neglect patients, Vuilleumier et al., 2004 or Priftis et al., 2006) to about 40 (Gevers et al., 2006c). The magnitude of the reported average SNARC slopes (*sl*) varies mostly between -10 ms (van Galen and Reitsma, 2008, adults group) and -3 ms (Fias et al., 2001, Exp. 1). The number of trial repetitions (*k*) varies typically between 10 (e.g., Bachot et al., 2005) and more than 60 trials (for EEG studies such as Keus et al., 2005) across studies. The standard deviations for single items (*sdR*) may also differ substantially across studies. For the purpose of simulating data from diverse groups, we chose to use a wide range of possible standard deviations, from small (similar to those reported by Nuerk et al., 2005), to medium (similar to reported by Cipora et al., 2009), to very large (as found from inspection of our own unpublished data, or characteristic of children see, e.g., van Galen and Reitsma, 2008).

#### Sample Size

Sample size (*n*) has a direct impact on the estimation of the standard error of the mean. While keeping all other factors constant, larger sample sizes imply smaller standard error and a larger probability of detecting a non-zero effect. In the present study, we compared modest (*n* = 10), typical (*n* = 20), large (*n* = 30) and very large (*n* = 40) sample sizes.

For power plot preparation, sample sizes between 10 and 20 were also simulated, because very often samples in SNARC studies fall between 10 and 20 participants (i.e., *n* = 12, 14, 16, and 18)^1^. We have also added sample size of *n* = 50. In Simulation 2, the sample size refers to one group and always assumes equal numbers of participants per group.

#### Slope Size

Slope size (*sl*) describes the absolute degree of association between number magnitude and response codes in milliseconds per magnitude unit. Keeping all other factors constant, the larger the slope size, the more easily detectable it is. In the present study, SNARC slopes in the range between -7 ms and -1 ms in steps of 2 ms were examined. For Simulation 2, between group differences (*sld*) ranging from large (±8 ms) to small (±2 ms) in steps of 2 ms were examined.

#### Number of Repetitions

The number of repetitions per item (*k*) affects the inclination of the individual SNARC slopes. With an increasing number of repetitions, the estimate of the individual SNARC slopes becomes more stable. When the number of repetitions per item is small, individual trials have a larger influence on the computation of the individual SNARC slopes. Consequently, the probability that the SNARC slope estimated in single participants may be contaminated is large and the measurement error is larger. For statistical analysis (in both simulations), four levels were chosen (*k* = 10, 20, 30, 40). For power plots, we additionally used *k*-values up to 120 increasing in steps of 10.

#### Intra-individual Variability

Depending on the overall speed and accuracy as well as on transient influences on performance (e.g., boredom or tiredness) responses to single items may vary dramatically. Estimation of the SNARC slope in participants who respond in a very inconsistent way is much more variable than in participants showing very consistent performance. A simple measure of performance consistency is the average standard deviation observed within-condition (SD response; *sdR*). In the present study, four values were chosen (*sdR* = 75, 150, 225, and 300 ms). To produce realistic estimations of reaction times distributions, intra-individual variability was modeled using a lognormal distribution with a logarithmic compression of σ = 0.5^2^.

### Simulation 1: Detecting an Existing SNARC Effect

In this simulation, the power to detect an existing SNARC effect was investigated.

Response times for left and right hand were generated using Equation 1, where “RT” indicates reaction time, “magnitude” the different number magnitudes, “slope” the difference in reaction time associated with increasing number magnitude, and “error” for random noise. The error term followed a log-normal distribution with mean equal to 0 and standard deviation equal to the intra-individual variability defined as above and multiplied by a term of logarithmic compression, σ = 0.5. To simplify the model, in all simulations the same intercept for both hands was assumed^3^. Right hand RTs were modeled with slopes of decreasing values while left hand RTs were modeled with slopes of ascending values.

RT=magnitude*slope/2+error

Values obtained from Equation 1 were averaged^4^ across all replications of each single number magnitude. Subsequently, values obtained for the left hand were subtracted from those obtained for the right hand, yielding the difference in reaction times dRT (Fias et al., 1996). The dRTs were regressed on number magnitude using an ordinary least squares regression model. SNARC slopes were obtained for each simulated participant and then averaged, thereby yielding the average SNARC slope for each simulated sample. We simulated reaction times for numbers 0–9. Therefore, individual slopes were calculated using 10 data points. However, researchers often use different number sets (e.g., 8 numbers: 1–9 excluding 5). As results of the linear regression largely depend on the number of data points that are used in fitting the slope, in Supplementary Material 1 we calculated similar simulations based on different stimulus sets and briefly discuss obtained results.

### Simulation 2: Detecting an Existing Difference in Slopes

In this simulation, the power to detect a group difference was assessed. In each simulation, SNARC slopes were obtained for two independent groups in each run. Estimates of individual SNARC slopes were obtained in the same way as in Simulation 1, but for the two groups separately. Group differences were computed and tested inferentially.

### Data Analysis

#### Simulation 1: Detecting a SNARC Effect

Each observation in the final data set can be interpreted as the result of a SNARC experiment in which the average SNARC slope was compared with 0 using a one-sample *t*-test (one-sided). The *p*-values associated with these *t*-tests were coded as 1 when the *p*-value was smaller than 0.05 and 0 when not (using the source code provided, the reader may conduct analyses considering more conservative alpha levels, e.g., 0.01 and 0.001 as well). Power tables considering these more conservative alpha levels can be found in Supplementary Material 2). To examine which factors influence the proportion of significant SNARC slopes as well as to find possible interactions between them, an ANOVA model was calculated with a 4 [Number of repetitions (*k*): 10, 20, 30, 40] × 4 [Assumed magnitude of SNARC slope (*sl*): -7, -5, -3, -1 ms] × 4 [Sample size (*n*): 10, 20, 30, 40] × 4 [SD response (*sdR*): 75, 150, 225, and 300 ms] between-subjects design. Thus, the total number of experimental conditions was 256. Each one of these conditions was replicated 1000 times. All simulations and statistical analyses were conducted in the software R (R Core Team, 2016; the source code^5^.

#### Simulation 2: Detecting a Difference between Groups

Each observation in the final data set can be interpreted as the result of a SNARC experiment in which average SNARC slopes obtained from two independent groups of participants were compared using independent-samples *t*-test (two-sided). The *p*-values associated with these *t*-tests were coded as 1 when the *p*-value was smaller than 0.05 and 0 when not. Again, a 4 [Number of repetitions (*k*): 10, 20, 30, 40] × 4 [Assumed difference in SNARC slopes (*sld*): 8, 6, 4, 2 ms] × 4 [Sample size (*n*): 10, 20, 30, 40] × 4 [SD response (*sdR*): 75, 150, 225, and 300 ms] between-subjects design ANOVA was used. The total number of experimental conditions was 256. Each one of these conditions was replicated 1000 times. All simulations and statistical analyses were conducted in the software R (the source code)^5^.

## Results

Since highly significant effects were expected regarding most factors, effect size estimates, partial eta^2^ ($\eta_{p}^{2}$) were chosen as a measure of the relevance of simulated parameters complementing the *F*-test significance level (MacCallum et al., 1999). Only tests presenting at least a small effect size ($\eta_{p}^{2}$ > 0.01, Cohen, 1988) will be interpreted. The following criteria for quantifying effect sizes were adopted: 0.01 ≤ small effects < 0.06 ≤ moderate effects < 0.14 ≤ large effects (Cohen, 1988). Proportion of significant to non-significant results in each condition represents the expected power to detect a SNARC effect and group differences in that condition which is presented in power plots, and also available in a form of a table in Supplementary Material 2.

### Simulation 1

When SD response is large, even a large SNARC effect may be hard to detect (**Table 1**). In that case, the only way to ensure at least satisfactory power to detect SNARC seems to be to employ large sample sizes and ask participants to perform more than a typical number of repetitions (*k* > 20). Since one expects a large SD response, a sample size of *n* > 20 seems to be a minimal design requirement. The large main effect of slope size reflects the fact that different slope sizes were investigated (-7 to -1 ms). More interesting, a moderate but robust main effect of the number of repetitions indicates that a larger number of repetitions leads to larger SNARC effect estimates. The same finding holds regarding the moderate effect of sample size: the power to detect an existing SNARC effect increases with sample size. A small effect of the interaction *sl* × *sdR*, shows that with increasing intra-individual variability, the decrease in power is much larger for small and moderate slopes (-1 and -3 ms). The small effect of the interaction *k* × *sl* × *sdR*, indicates that a drop in power for small SNARCs is substantial when SD response rises from small to moderate. With an increase in SD response, it is hard to obtain optimal power even by increasing the number of repetitions. Moreover, the small effect of the interaction *sl* × *n* × *sdR*, indicates that the most substantial drop of power occurs between small and moderate levels SD response, *sdR* = 75 ms and *sdR* = 150 ms, whereas the drop between large and very large levels of SD response is less pronounced.

**Table 1:** Statistics regarding the detection of an existing SNARC.

Effect	*df*	SS	MS	*F*^∗^	$\eta_{p}^{2}$	Effect
Number of repetitions (*k*)	3	1546	515	5162	0.06	Moderate
Slope size (*sl*)	3	16707	5569	55796	0.40	Large
SD Response (*sdR*)	3	4681	1560	15635	0.16	Large
Sample size (*n*)	3	2006	669	6700	0.07	Moderate
Number of repetitions (*k*) × Slope size (*sl*)	9	150	17	167	0.01	Small
Number of repetitions (*k*) × SD Response (*sdR*)	9	118	13	131	0.01	Small
Number of repetitions (*k*) × Sample size (*n*)	9	31	3	34	0.00	Negligible
Slope size (*sl*) × SD Response (*sdR*)	9	1232	137	1371	0.05	Small
Slope size (*sl*) × Sample size (*n*)	9	194	22	216	0.01	Small
Sample size (*n*) × SD Response (*sdR*)	9	182	20	202	0.01	Small
Number of repetitions (*k*) × Slope size (*sl*) × SD Response (*sdR*)	27	564	21	209	0.02	Small
Number of repetitions (*k*) × Slope size (*sl*) × Sample size (*n*)	27	136	5	50	0.01	Small
Number of repetitions (*k*) × Sample size (*n*) × SD Response (*sdR*)	27	29	1	11	0.00	Negligible
Slope size (*sl*) × Sample size (*n*) × SD Response (*sdR*)	27	700	26	260	0.03	Small
Number of repetitions (*k*) × Slope size (*sl*) × Sample size (*n*) × SD Response (*sdR*)	81	223	3	28	0.01	Small
Residuals	255744	25525				


*^∗^*p*-values for all effects <0.001 therefore not reported in the table.*

**FIGURE 1:**
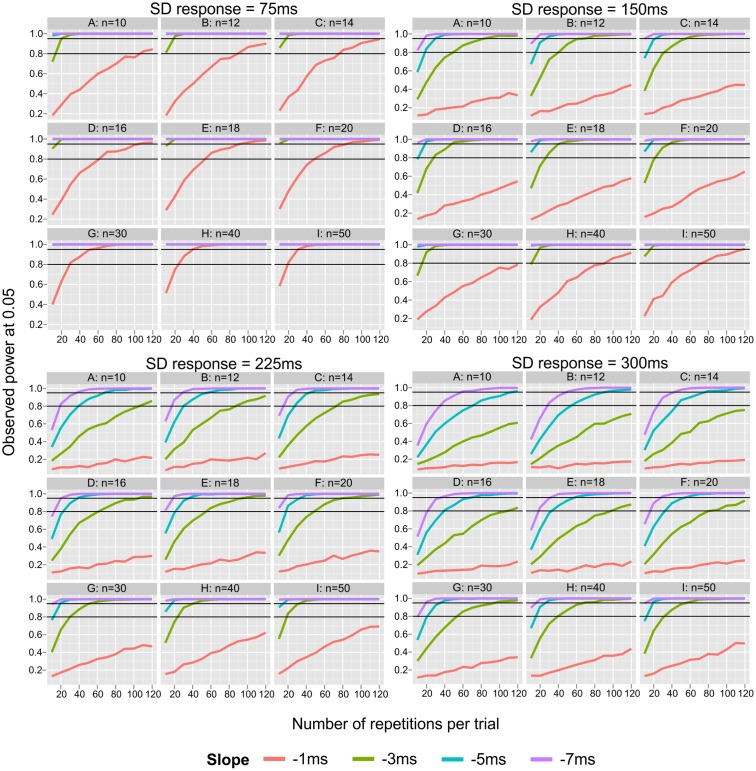
Power graph for all parameters used in the simulations (power of detecting existing SNARC effect at 0.05). Small panels represent sample sizes, big panels represent levels of intra-individual variability (SD response). *X* axis represents number of repetitions from 10 to 120 in steps of 10. Black lines represent powers of 0.80 and 0.95.

**Figure 1** depicts the effect of number of repetitions and sample size on the power to detect an existing SNARC effect depending on SD response (note that it covers a wider range of sample sizes and number of repetitions than included in the ANOVA). **Figure 1** reveals that power is largely insufficient to detect a very small SNARC effect (i.e., *sl* = -1 ms) when considering the typical number of repetitions (10 < *k* < 40) and sample sizes (10 < *n* < 40). Moreover, detailed inspection of the top rows of each panel shows that when sample sizes are small, even a typically sized SNARC effect of -3 ms remains largely undetected. Under these conditions, a large SNARC slope of -5 ms can be detected with power >0.95 when the number of repetitions of each item is smaller than 30 (and SD response is not very large, *sdR* ≤ 225 ms).

When sample size is *n* > 14, the power to detect an existing SNARC effect of typical size (i.e., *sl* ≤-3 ms) is almost always larger than 0.8 (excluding large and very large SD response, *sdR* < 300 ms). When sample sizes are large (*n* > 40) the observed power is over 95% for SNARC slopes of typical size (i.e., *sl* ≤-3 ms). Finally, the power to detect large and very large SNARC slopes (in the case of small to moderate SD response, *sdR* ≤ 150 ms) is highly satisfactory for every sample size *n* > 12.

A more detailed analysis of the effect of the number of repetitions per item on the probability to detect the SNARC effect indicates that the power to detect a very small SNARC effect (i.e., *sl* = -1 ms) is largely insufficient even when a very large number of repetitions per item is employed. Moreover, detailed inspection of top panels of **Figure 1** shows that when sample sizes are small, the power to detect a typical SNARC effect of -3 ms increases substantially with the number of repetitions per item. Under these conditions, a large SNARC slope of -5 ms can be detected with a power below 0.95, when the number of repetitions of each item is smaller than 30.

When sample size is *n* > 14, the power to detect an existing SNARC effect of typical size (i.e., *sl* ≤-3 ms) is almost always larger than 0.8 (assuming small or moderate SD response). When sample sizes are large (*n* = 40) the observed power is more than 0.95 for SNARC slopes of typical size (i.e., *sl* ≤-3 ms). Finally, the power to detect large and very large SNARC slopes is highly satisfactory for every sample size larger than 14.

### Simulation 2

**FIGURE 2:**
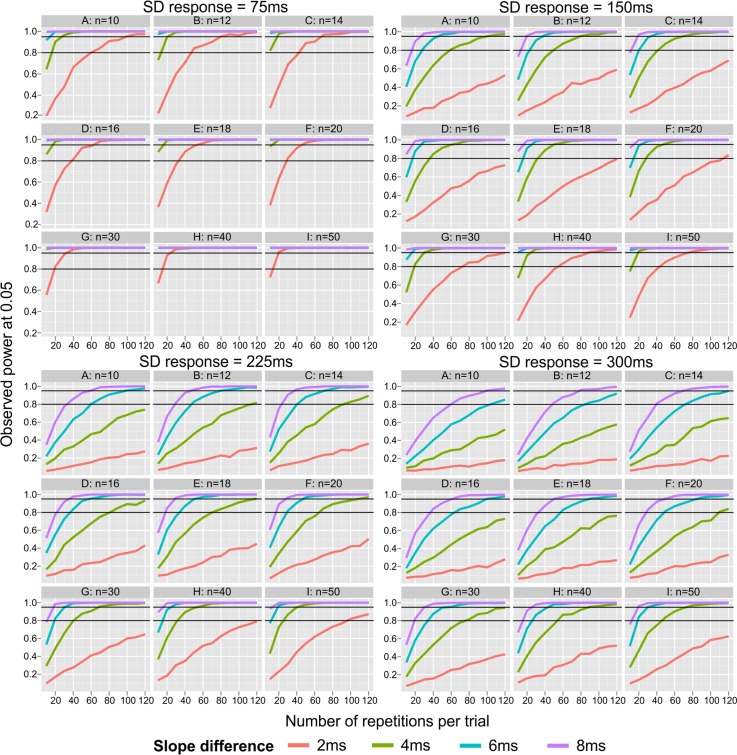
Observed power for detecting an existing difference between two SNARC slopes (at 0.05 level). Small panels represent sample sizes, big panels represent levels of intra-individual variability (SD response). *X* axis represents number of repetitions from 10 to 120 in steps of 10. Black lines represent 0.80 and 0.95 power.

In Simulation 2, the probability to detect an existing difference between two SNARC slopes was estimated. As depicted in **Table 2**, the size of the difference and intra-individual variability had large effects on the ability to detect an existing group difference in SNARC slopes. Notably, the effect of intra-individual variability was almost the same as the effect of actual between-group differences. Moreover, the number of repetitions and the sample size had a moderate impact on the detection of the SNARC effect. The interaction *sld* × *sdR* had a small effect on power. In the case of a very small slope difference (*sld* = 2 ms), the decline in power is much more pronounced with increasing SD response from very small (*sdR* = 75 ms) to moderate (*sdR* = 150 ms). Large (*sld* = 6 ms) and very large (*sld* = 8 ms) slope differences are much more resistant against an increase in SD response (**Figure 2**). The small effect of the interaction, *k* × *sld* × *sdR*, indicates the most substantial decrease of the power to detect a moderate slope difference (*sld* = 4 ms) between small (*sdR* = 75 ms) and moderate (*sdR* = 150 ms) SD response. The small effect of the interaction, *sld* × *n* × *sdR*, shows a robust increase in power between *n* = 10 and *n* = 20, especially for moderate (*sld* = 4 ms) and big (*sld* = 6 ms) slope differences in the case of moderate (*sdR* = 150 ms) and large (*sdR* = 225 ms) SD response.

**Table 2:** Statistics regarding the detection of an existing difference between SNARC slopes.

Effect	*df*	SS	MS	*F*^∗^	$\eta_{p}^{2}$	Effect
Number of repetitions (*k*)	3	2495	832	7447	0.08	Moderate
Slope difference (*sld*)	3	11491	3830	34293	0.29	Large
SD Response (*sdR*)	3	8983	2994	26807	0.24	Large
Sample size (*n*)	3	2879	960	8592	0.09	Moderate
Number of repetitions (*k*) × Slope difference (*sld*)	9	62	7	61	0.00	Negligible
Number of repetitions (*k*) × Sample size (*n*)	9	18	2	17	0.00	Negligible
Number of repetitions (*k*) × SD Response (*sdR*)	9	191	21	190	0.01	Small
Slope difference (*sld*) × Sample size (*n*)	9	81	9	81	0.00	Negligible
Slope difference (*sld*) × SD Response (*sdR*)	9	1266	141	1260	0.04	Small
Sample size (*n*) × SD Response (*sdR*)	9	213	24	212	0.01	Small
Nuamber of repetitions (*k*) × Slope difference (*sld*) × Sample size (*n*)	27	91	3	30	0.00	Negligible
Number of repetitions (*k*) × Slope difference (*sld*) × SD Response (*sdR*)	27	707	26	234	0.02	Small
Number of repetitions (*k*) × Sample size (*n*) × SD Response (*sdR*)	27	88	3	29	0.00	Negligible
Slope difference (*sld*) × Sample size (*n*) × SD Response (*sdR*)	27	771	29	256	0.03	Small
Number of repetitions (*k*) × Slope difference (*sld*) × Sample size (*n*) × SD Response (*sdR*)	81	301	4	33	0.01	Small
Residuals	255744	28566				


*^∗^*p*-values for all effects <0.001 therefore not reported in the table.*

**Figure 2** depicts the effect of number of repetitions, sample size and SD response on the power to detect a difference between two independent groups regarding SNARC slopes (note that it covers a wider range of sample sizes and number of repetitions than included in the ANOVA). It reveals that the power is largely insufficient to detect a small difference between groups (i.e., *sld* = 2 ms), when considering the typical number of repetitions (10 < *k* < 40), particularly when the SD response is above the smallest level (*sdR* > 75 ms). Interestingly, when both sample sizes and number of repetitions are sufficiently large, 0.80 power can be reached even for a small between-groups differences, assuming small (*sdR* = 75 ms) or moderate (*sdR* = 150 ms) SD response. Moreover, detailed inspection of top rows of each panel shows that when sample sizes are small (*n <* 16), a moderate difference between groups of 4 ms cannot be detected even when the number of repetitions is sufficiently large (*k* = 40). Finally, a large group difference of 6 ms can be detected with power above 0.80 when the number of repetitions of each item is larger than 30, irrespective of SD response.

When sample sizes *n* > 16, the power to detect an existing group difference of typical size (i.e., *sld* ≥ 4 ms) is almost always larger than 0.80. Finally, the power to detect large and very large differences between groups is highly satisfactory for every sample size larger than 16. The examination of group differences seems to accomplish the necessary power when sample sizes are larger than *n* > 30 per group, whereas the number of repetitions per item should be larger than *k* > 40 only when *sdR* ≤ 225 ms (*n* > 20 and *k* > 20 for smaller *sdR*). In the case of a higher SD response, design requirements are much more demanding.

## Discussion and Conclusion

In the present study, the empirical power to detect an existing SNARC effect and to detect a group difference in SNARC slopes were examined using Monte Carlo simulations. Inspection of the results of Simulation 1 reveals that both sample size and number of repetitions as well as intra-individual variability determine the probability to detect an existing SNARC effect. In general, power problems are evident when sample size and number of repetitions per item are smaller than 20. Therefore, the results reveal that typically design parameters for investigating SNARC may systematically lack statistical power. Moreover, Simulation 2 revealed that sample size and number of repetitions as well as their interaction with the size of the difference in SNARC slopes determine the power to detect group differences. Notably, in both cases the intra-individual variability in response latencies has a large impact on the power to detect SNARC, and even more pronounced – to detect between-group differences. While sample size and number of repetitions are under direct control of the experimenter when planning the experimental design, intra-individual variability is an intrinsic variable characteristic of the participants being tested and therefore more difficult to manipulate. Finally, a comparison of the two simulations reveals a stronger effect of sample size and number of repetitions on the probability of detecting group differences when compared to detecting a SNARC slope. In the following, these results will be discussed in more detail.

### Slope Size

The size of the SNARC slope had the largest impact on the probability to detect a SNARC effect. This appears to be a trivial result, since the differences were inserted in the simulations by the authors themselves, but it is not. These results reveal the need to obtain a good *a priori* estimate of the size of the SNARC slope to reach the correct decision about other aspects of the design, such as sample size and the number of repetitions per item. In Simulation 1 SNARC slopes in the range between -1 ms and -7 ms were evaluated. Although a SNARC slope of -1 ms can be considered a low value for most investigations on the SNARC effect, slopes of around -3 ms have been reported quite often in the literature [e.g., Fias et al., 2001, Exp. 1 (-2.03 ms); Ito and Hatta, 2004 (-2.52 ms; -3.04 ms; -2.57 ms for Exp. 1, 2a, 2b, respectively); Nuerk et al., 2005 (-3.79 ms); Gevers et al., 2006c (-4.21 ms); Shaki and Fischer, 2008, Exp. 1, “Hebrew” condition (-4.43 ms)]. Therefore, these values can be considered a reference both for the interpretation of the present simulations as well as a parameter for a “typical SNARC slope” in further studies. Moreover, the size of the SNARC slope difference had the largest impact on the probability to detect a difference between two groups. Inspection of the literature on differences between groups reveals a considerable number of studies reporting (non-significant) differences between groups in the range between 1 ms (Castronovo and Seron, 2007, Exp. 1a and 1b) and more than 10 ms (Fischer and Rottmann, 2005; Priftis et al., 2006; van Galen and Reitsma, 2008). In the present study, differences between groups in the range of 2–8 ms were examined. Although a SNARC slope of -2 ms may seem to be small, a difference of 2 ms between two groups may have an important theoretical impact.

Finding a reliable estimate of the size of the SNARC effect may be a hard task. As shown in a meta-analysis (Wood et al., 2008) the size of the SNARC effect is heterogeneous and depends on several design parameters such as task, stimulus, response mode, and age of participants. All of these factors seem to determine the size of the SNARC effect one can expect when designing new studies. Due to these difficulties, when adopting experimental manipulations barely or never tested before, it is desirable to adopt a conservative estimate of the SNARC effect size (i.e., -3 ms to -4 ms) as a reference for the selection of further design parameters with the view of optimizing power. However, when the upcoming experiment replicates many parameters from previous studies in which a larger SNARC effect (i.e., -5 ms to -7 ms) was found, experimenters may feel confident to select more liberal parameters for the upcoming study (smaller sample sizes, smaller number of repetitions).

The same design parameters regulating the power to detect an existing SNARC effect also apply to the detection of group differences. Since the number of studies examining group differences is still relatively small, the estimation of differences may sometimes raise the impression of blind guessing. Under such circumstances, it is desirable to assume that group differences will not be particularly large and design the study accordingly. If the experimenter assumes that a group difference will be moderate, an experimental design with 20 participants per group vs. 20 repetitions may be indicated since it reaches 95% power to detect an existing group difference of 4 ms in the SNARC slopes (**Figure 2**). Slope size also showed interactions with sample size and number of repetitions per item. The relevance of these interactions will be discussed in the next sections below.

### The Effect of Intra-individual Variability

Intra-individual variability had a dramatic impact on the power of detecting a SNARC effect in the present study. Therefore, together with estimating expected magnitude of the SNARC effect, one must consider expected intra-individual variability in the examined sample. Many factors may influence this kind of variability. First of all, characteristics of the sample (i.e., examining children or elderly participants or even neurologic patients, one may expect bigger variability than in the case of examining young adults). Furthermore, the level of participants’ motivation may influence the variability (i.e., more motivated subjects may be more regular in their responses). The design of the whole experiment may influence intra-individual variability as well. Rarely the experiment consists only of a single condition of the parity judgment task. Normally participants are asked to perform some more procedures. Therefore, when they are tired (and or) bored, variability in their RTs may increase. Here, we present simulations with parameters set from very small variability to very high. Having no cues that intra-individual variability may be extraordinary, one may assume that it would be relatively low (ca. 150 ms). Designing the experiment, one can inspect the power plots (or supplementary power tables in Supplementary Material 2) and look at the desired parameters for a given SD of response level.

Luckily, bigger variability in response latencies is associated with bigger SNARC slopes *r* = -0.37 (Cipora and Nuerk, 2013). Intra-individual variability is highly correlated with overall reaction time (usually *r* > 0.80; see Cipora et al., 2016), which has been shown to be negatively correlated with magnitude of SNARC as well (Gevers et al., 2006c). Therefore, with bigger intra-individual variability, one may expect bigger SNARC slopes than when variability is small.

Moreover, increasing the number of repetitions may help to decrease intra-individual variability in reaction times (and decrease the influence of outlier reaction times on aggregated averages). Therefore, increasing the number of repetitions may lead to a twofold increase in the power to detect SNARC (i.e., directly increasing power by obtaining better estimations of mean reaction times, and indirectly by decreasing intra-individual variance in response latencies).

The impact of intra-individual variability was particularly visible in the detection of group differences (Simulation 2). This result can be explained by the fact that in the case of detecting SNARC (Simulation 1), an increase of intra-individual variability only affects the variance in one group (and the criterion value in a one-sample *t*-test has no variance at all); while in detecting between-group differences, increasing intra-individual variability raises the variance in both groups. Moreover, intra-individual variability interacted with slope size (Simulation 1). The drop in power observed when intra-individual variability increases was much bigger for small and moderate slope sizes.

### Sample Size

Sample size has a moderate impact on the probability of detecting a SNARC effect and a group difference. Moreover, sample size and slope size interact in both simulations. When sample sizes are below 20, only large SNARC effects can be reliably detected. This is due to the fact that the most substantial increase in power is obtained by increasing sample sizes up to *n* = 20 (**Figure 1**). Below this range, typical SNARC slopes of about -3 ms remain largely undetected, thereby jeopardizing theory building. Beyond the range of *n* = 20, power only increases slowly and at a high cost with an increment in sample size. For instance, assuming that the number of repetitions per stimulus is fixed at 10, and the true SNARC slope is around -3 ms, 95% power can be reached only by increasing sample size to at least 40 participants (but only on the condition of minimal intra-individual variability; for bigger intra-individual variability, the sample size would have to be increased to more than 50 participants).

In that case, increasing the number of repetitions should be considered instead. Nevertheless, having 20 participants performing 20 repetitions seems to be a reasonable solution in most cases. When testing group differences, increasing sample sizes above 20 per group is particularly laborious, since in this case sample size = *n* × 2. Therefore, studies on group differences are more economical when individual participants solve more trials, since the effort to build a large sample is twice as large as in within-subject studies.

### Number of Repetitions per Item

The third largest effect obtained in Simulations 1 and 2 was the effect of the number of repetitions. Inspection of **Figures 1**, **2** reveals that even effects that are only moderate or small in size still may present dramatic differences in power. In a similar fashion as for sample size, a small number of repetitions (*k* = 10) leads to a large power drop. Ironically, a large number of previous studies have employed a small number of repetitions per item in the range between *k* = 10 and *k* = 15 repetitions (*k* = 10: Bachot et al., 2005; Fischer and Rottmann, 2005; Nuerk et al., 2005; Shaki and Petrusic, 2005; *k* = 12: Vuilleumier et al., 2004; *k* = 13: Priftis et al., 2006; *k* = 15: Gevers et al., 2006a; Castronovo and Seron, 2007). Considering that most studies on the SNARC effect have employed a number of repetitions in this rage, one may argue to what extent this has contributed to a systematic underestimation of the presence of the SNARC effect as well as the existence of group differences.

Moreover, the 3-way interaction among Number of Repetitions × Slope size × SD response shows that for small intra-individual variability, the number of repetitions is not very important (partially due to a ceiling effect) but with increasing SD response, it becomes more important, and the effect of slope size on power increases. Moreover, these interactions also show that although sample size has a large effect on the probability to detect both a SNARC effect as well as a difference between groups, simply increasing sample sizes is not always sufficient for detecting an existing SNARC effect. In fact, in several cases this is the less expedient way to improve the quality of an experiment. Fortunately, increasing the number of repetitions to around 20 seems to mitigate most problems with the detection of a SNARC effect, assuming that sample size is not smaller than 20 participants. One must keep in mind that in most cases, the size of intra-individual variability should decrease substantially with the increase of trials in the task, therefore additionally improving the odds to successfully detect an existing SNARC effect.

### Final Considerations

To summarize our results in one sentence, one can say that SNARC experiments should always test at least 20 persons in each group, and present each stimulus at least 20 times per condition, to have a fair chance to detect the presence of the SNARC effect as well as group differences in the effect. This rule-of-thumb contributes to the investigation of the SNARC effect by making explicit that there are minimal design requirements necessary for detecting differences between groups, which have not always been met in studies published to date. The 20^∗^20 rule has a practical use. For instance, a parity judgment task using 10 different stimuli, each one being presented 20 times, takes a relatively short time (about 12 min) and the increase in power is substantial in comparison with a standard paradigm of 10 repetitions per item. This guideline holds true in most cases; nevertheless, when working with groups with high intra-individual variability, one should consider a more conservative approach (i.e., more participants and more repetitions).

**Figures 1**, **2** (also available in a form of a table in Supplementary Material 2) provided in the present study may help future SNARC investigators to design more economic and efficient designs, which have the necessary power to detect existing SNARC slopes as well as differences between groups. To make use of these figures, the only requirement is to obtain a reasonable estimate of the size of the SNARC slope from the existing literature and the expected intra-individual variability in response latencies. Then, one must define the lowest level of power acceptable for the study and choose how many repetitions per item and participants are necessary/feasible.

A short inspection of the experimental parameters employed in the recent literature suggests chronic power deficits when trying to detect a SNARC effect^6^ or compare the relative size of SNARC slopes across different groups of participants. However, since the interest in the meaning and the diagnostic value of the SNARC effect is increasing, researchers should be adequately equipped to detect not only a significant SNARC effect but also group differences. As we have shown with Monte Carlo simulations, the number of repetitions per item is as important for an efficient design as sample size, and needs to be taken into consideration when designing an SNARC experiment. Finally, the present study is a reference not only for research on the SNARC effect, but it includes all fields in which regression slopes are used to detect the presence of a cognitive representation or a group difference (e.g., mental rotation, see Böckler et al., 2011 or numerical distance effect see De Smedt et al., 2009).

Notably, our findings are in line with general recommendations regarding increasing power to detect main effects or interactions in ANOVA models. These general recommendations can be summed up as follows: (1) increasing between group (between condition) differences, (2) limiting within group (within condition) variance, and (3) testing sufficiently large samples. Importantly, power to detect interactions in ANOVA models is considerably smaller than power to detect main effects. Even more radical drops in power occur in case of higher-order interactions (Cohen, 1988). Our recommendation to increase the number of repetitions per trial aims at decreasing the variance caused by the error component associated with each particular reaction. The recommendation to test a sufficiently large sample size is self-evident. On the other hand, intra-individual variability in reaction times as well as slope size (differences in slopes) largely depends on a particular sample, and therefore one cannot directly influence them. Nevertheless, these factors need to be considered to obtain sufficient power.

Despite accessibility of these general recommendations about power, several methodological contributions show that a huge proportion of studies in the field of psychology are underpowered (e.g., Maxwell, 2004). Therefore, it seems important that apart from general, well-known recommendations, researchers working in a particular field are equipped with guidelines that are specifically tailored to the given experimental task. This seems particularly important in the case of the SNARC effect, as it is quantified by relatively complex calculation methods, which due to the differential nature of the dRT measure and consequently, requires summing up the error term.

Last but not least, it is worth mentioning that the power calculations of the present study apply equally to ANOVA designs as defined by Pinhas et al. (2012) and Tzelgov et al. (2013).

## Author Contributions

All authors listed have made a substantial, direct and intellectual contribution to the work, and approved it for publication.

## Conflict of Interest Statement

The authors declare that the research was conducted in the absence of any commercial or financial relationships that could be construed as a potential conflict of interest.

## Footnotes

1 Moreover, after a first run of simulations for sample sizes of 10, 20, 30 and 40 it turned out that differences in power between sample sizes of 10 and 20 were dramatic.

2 To check whether this definition of the error term fits the data properly, we reanalyzed some of our SNARC data published elsewhere (Cipora et al., 2016). We selected randomly 10 participants who participated in this experiment, and tested whether the residuals from the typical SNARC regression (i.e., dRT regressed on number magnitude) deviated significantly from the lognormal distribution using the one-sample Kolmogorov–Smirnov test. In case of 8 out of 10 participants, the test did not indicate significant deviations. We considered this evidence for the use of the lognormal distribution as sufficient for our purposes.

3 Using the same intercepts (i.e., assuming the same mean RT for both hands) is not an oversimplification, because results regarding differences in reaction times between hands are inconsistent. Dehaene et al. (1993), Fias (2001) and Cipora et al. (2011) report a highly significant right hand advantage. Bächtold et al. (1998) and Nuerk et al. (2005) report no difference in reaction times between hands. Gevers et al. (2006b) report a marginally significant left hand advantage while Herrera et al. (2008, Exp. 2) report a highly significant left hand advantage. Moreover, while regression slopes are calculated, a relatively small difference in reaction times between hands seems to influence only the intercept of the regression line, not its weight.

4 By modifying the code “Varied_intervals_extended_simulation_1.R” provided on https://osf.io/ud2cr/, in a place we marked with a comment, one may also use medians instead of means to aggregate the data.

5 https://osf.io/ud2cr/

6 But see important exceptions such as Keus et al. (2005), Gevers et al. (2006a,b,c), Notebaert et al. (2006), and Schwarz and Müller (2006), where very good power levels were reached.
