# Supplementary material for: Finding the SNARC Instead of Hunting It: A 20∗20 Monte Carlo Investigation
Source: Front Psychol. 2017 Jul 18;8:1194. doi: 10.3389/fpsyg.2017.01194 (PMC5513957; doi:10.3389/fpsyg.2017.01194)
Supplement: Supplementary file 1 [file Data_Sheet_1.pdf]

Cipora K and Wood G (2017) Finding the SNARC Instead of Hunting It: A 20*20 Monte Carlo Investigation. *Front. Psychol. 8*:1194. doi: 10.3389/fpsyg.2017.01194

SUPPLEMENTARY MATERIAL 1

Additional analyses including different number intervals and proportion of participants revealing negative slopes

Improved code (also available on https://osf.io/ud2cr/) allows for estimating the power to detect the SNARC effect depending on which numbers are used to calculate individual regression slopes. Six different intervals were used: (1) all numbers from 0-9; (2) numbers 1, 2, 8 and 9; (3) numbers 1, 4, 6 and 9; (4) numbers 1-9 excluding 5; (5) numbers 1-4; (6) numbers 1-8. Additionally, it calculates the proportion of participants within each simulated experiment who revealed negative slopes. Furthermore, it allows for estimating SNARC slopes based on median reaction times instead of mean reaction times. Here we summarize most important findings regarding role of number interval and proportion of participants revealing negative SNARC slopes.

Number interval

In this additional analysis we checked whether the number of points one uses for individual regression (i.e., the stimuli set used in the experiment) influences the power to detect the SNARC effect. The same parameters were used as those reported in the main text. Results were submitted to 6 [interval (*int*): 0-9; 1, 2, 8, 9; 1, 4, 6, 9; 1-9out5; 1-4; 1-8] × 4 [repetitions (k): 10, 20, 30, 40] × 4 [slope (*sl*): -7; -5; -3; -1] × 4 [sample size (*n*): 10, 20, 30, 40] × 4 [SD Response (*sdR*): 75; 150; 225; 300] ANOVA with presence of the SNARC effect at alpha level

.05 as the dependent variable. Effect sizes using η_p^2 were used to quantify these influences. Again, we interpreted only effects with corresponding effect sizes ≥ .01.

There was a robust effect of interval (η_p^2 = .13). The lowest power was observed in the case of interval 1-4 (.28). Importantly there was no huge difference between interval 0-9 and 1-9 excluding 5 (.70 and .65 respectively). The power for the 1-8 interval was lower (.60), suggesting that when one wants to exclude 0 (as recommended by Nuerk et al., 2004) it is better to use numbers 1-9 and exclude 5 than the interval 1-8 (moreover it allows keeping number parity and magnitude orthogonal). For numbers 1, 2, 8 and 9 the power was larger than for numbers 1, 4, 6, 9 (.62 and .56 respectively). This suggests that in case only 4 numbers are to be used, it is better to use the 1, 2, 8, 9 set (despite the fact that in both cases parity and magnitude are orthogonal).

A meaningful effect size (≥ .01) was present only in the case of an *int* × *sl* interaction (η_p^2 = .02). With a slope of -1, the intervals did not differ much in power to detect the SNARC (and the power was constantly very low). In case of other slopes, the intervals were much more differentiated, but the pattern of relation between interval and power to detect the SNARC was virtually the same. All other interactions of interval were accompanied by negligible effect sizes (all $\eta_p^2 \leq .007$).

Nevertheless, these results must be taken with caution as the SNARC effect seems to be present when varied number intervals are used, and depends more on the relative value of numbers used in the task than on their absolute magnitudes (e.g., Dehaene et al., 1993; Exp. 3).

Proportion of participants revealing negative slopes

Typically studies show that around 70% of individuals reveal negative slopes (Wood et al., 2006). Having uncovered that the power to detect SNARC depends on several parameters as

discussed in this paper, we wanted to see whether the proportion of participants revealing negative slopes would change depending on the task parameters as well. What was particularly interesting here was whether it would increase with an increasing number of repetitions. We investigated this by means of a 6 [interval (*int*): 0-9; 1, 2, 8, 9; 1, 4, 6, 9; 1-9out5; 1-4; 1-8] × 4 [repetitions (k): 10, 20, 30, 40] × 4 [slope (*sl*): -7; -5; -3; -1] × 4 [sample size (*n*): 10, 20, 30, 40] × 4 [SD Response (*sdR*): 75; 150; 225; 300] ANOVA with the proportion of participants revealing negative slopes as the dependent measure. Effect sizes (η_p^2) were used to quantify these influences. Again, only effect sizes ≥ .01 are discussed. Importantly, there was a strong effect of repetitions (η_p^2 = .11). The proportion constantly increased with increasing number of repetitions. The highest increase was observed between 10 and 20 repetitions. There was a meaningful and large effect of interval (η_p^2 = .22). The highest proportion was observed in the case of interval 0-9, followed by 1-9 excluding 5. The third largest proportion was observed in the case of numbers 1, 2, 8, 9 followed by numbers 1-8 and 1, 4, 6, 9. The smallest proportion was observed for the 1-4 interval. The main effect of the slope was also characterized by a very large effect size (η_p^2 = .45). Unsurprisingly, the proportion of participants revealing negative slopes increased radically with increasing slope. Similarly, there was a meaningful effect of SD response (η_p^2 = .36). The proportion decreased considerably with increasing *sdR*. The drop was largest between *sdR* levels of 75 and 150 ms.

There were also four meaningful two-way interactions. An *int* × *sl* (η_p^2 = .04) interaction indicated that the proportion of participants revealing negative slopes deviated more strongly depending on interval in the case of larger slopes, and there was virtually no variability in the case of the smallest slope (-1ms). The *int* × *sdR* interaction was also meaningful (η_p^2 = .02). Differences depending on interval were much more pronounced in

the case of smaller *sdR* values. A meaningful $k \times sl$ interaction ($\eta_p^2 = .01$) revealed that a larger increase in proportion to an increasing number of repetitions was present in the case of the strongest SNARC effects. The interaction $sl \times sdR$ ($\eta_p^2 = .06$) revealed that strongest drop in the proportion of participants revealing negative SNARC was present in the case of smaller *sdR* values. All other interactions were not meaningful ($\eta_p^2 \leq .006$).

These results could guide further investigations regarding interindividual variability in the SNARC effect. The data available to date are largely inconclusive with respect to whether an increasing number of repetitions increases the proportion of participants revealing negative slopes. Hoffmann et al. (2014) as well as Georges et al. (2016) used 9 repetitions and respectively 92 and 82 percent of participants revealed negative slopes. Nuerk et al. (2005) used 10 repetitions and found around 65% of negative slopes. Cipora & Nuerk (2013) used 20 repetitions and found that 71 percent of participants revealed negative slopes (i.e. close to typical score). Cipora et al. (2016) used 30 repetitions, and in groups that revealed a significant SNARC effect, the proportion of participants revealing negative slopes was 87 percent.

REFERENCES

Cipora, K., Hohol, M., Nuerk, H.-C., Willmes, K., Brożek, B., Kucharzyk, B., et al. (2016). Professional mathematicians differ from controls in their spatial-numerical associations. *Psychol. Res. 80*, 710–726. doi: 10.1007/s00426-015-0677-6

Cipora, K., and Nuerk, H.-C. (2013). Is the SNARC effect related to the level of mathematics? No systematic relationship observed despite more power, more repetitions and more direct assessment of arithmetic skill. *Q. J. Exp. Psychol. 66,* 1974–1991. doi: 10.1080/17470218.2013.772215

Dehaene, S., Bossini, S., and Giraux, P. (1993). The mental representation of parity and number magnitude. *J. Exp. Psychol. Gen. 122,* 371–396. doi: 10.1037/0096-3445.122.3.371

Georges, C., Hoffmann, D., and Schiltz, C. (2016). How math anxiety relates to number–space associations. *Front. Psychol. 7*:1401. doi: 10.3389/fpsyg.2016.01401

Nuerk, H.-C., Iversen, W., and Willmes, K. (2004). Notational modulation of the SNARC and the MARC (linguistic markedness of response codes) effect. *Q. J. Exp. Psychol. Sec. A 57*, 835–863. doi: 10.1080/02724980343000512

Nuerk, H.-C., Wood, G., and Willmes, K. (2005). The universal SNARC effect. *Exp. Psychol. 52*, 187–194. doi: 10.1027/1618-3169.52.3.187

Wood, G., Nuerk, H.-C., and Willmes, K. (2006). Variability of the SNARC effect: systematic interidividual differences of just random error? *Cortex 42,* 1119–1123. doi: 10.1016/S0010-9452(08)70223-5
